# Higher Bone Mineral Density in Obesity Masks Impaired Bone Remodeling and Disproportionate Musculoskeletal Adaptation in Young Women

**DOI:** 10.1096/fj.202603540R

**Published:** 2026-10-27

**Authors:** Yasmin Silva Forte, Caio Andrade‐Santos, Camila Pantoja‐Marinho, Luísa González‐Joaquim, Aline Penna‐De‐Carvalho, Paulo Roberto Falcão Leal, Karynne Grutter Lopes, Luiz Guilherme Kraemer‐Aguiar, Christina Barja‐Fidalgo

**Affiliations:** ^1^ Laboratory of Cellular and Molecular Pharmacology, Department of Cell Biology, IBRAG Universidade do Estado do Rio de Janeiro (UERJ) Rio de Janeiro Brazil; ^2^ Laboratory of Morphometry, Metabolism and Cardiovascular Diseases, Department of Anatomy, IBRAG Universidade do Estado do Rio de Janeiro (UERJ) Rio de Janeiro Brazil; ^3^ Obesity Unit (SAI‐Ob), Multiuser Clinical Research Center (CePeM) Pedro Ernesto University Hospital (HUPE), Universidade do Estado do Rio de Janeiro (UERJ) Rio de Janeiro Brazil

**Keywords:** bone formation, bone mineral density, bone quality, bone resorption, female, obesity, sarcopenia

## Abstract

Obesity is a multifactorial disease characterized by excessive adiposity that influences bone remodeling through increased mechanical loading, chronic low‐grade inflammation, and sedentary behavior. While obesity is linked to structural skeletal changes, its precise impact on bone mineral density (BMD) and bone remodeling in humans remains controversial, particularly in young women. This is particularly relevant because the majority of studies have investigated young women, in whom many of the observed skeletal alterations are attributable to aging rather than obesity per se. This study investigated the impact of increased adiposity on total, lumbar spine, and femoral neck BMD in 54 young Brazilian women with 18–40 years. Body composition and BMD were assessed by DXA, and plasma markers of bone formation and resorption were analyzed. Associations among adiposity, lean mass, BMD, and bone turnover markers were examined. Women with obesity exhibited higher total and regional BMD and greater lean mass than women without obesity. However, adjusting bone and lean mass for fat mass revealed a musculoskeletal imbalance. In addition, obesity was associated with a suppressed bone turnover profile; these women presented lower circulating levels of the formation markers P1NP and osteocalcin, alongside elevated resorption markers CTX‐I and RANK‐L/OPG. Ultimately, these findings demonstrate that bone remodeling is compromised despite an apparent increase in bone mass. Obesity in young Brazilian women is associated with higher BMD and lean mass; however, these findings may mask underlying detrimental alterations in bone remodeling. The observed imbalance between bone formation and resorption demonstrates that increased density does not reflect improved skeletal health, but rather compromised bone quality and structural fragility.

## Graphical abstract

Women with obesity exhibited higher total and site‐specific BMD and greater lean mass than lean controls. However, reduced BMD‐to‐fat and lean‐to‐fat mass ratios, increased CTX1 and RANK‐L/OPG, and decreased P1NP and osteocalcin indicated impaired bone remodeling, suggesting that increased BMD masks altered bone turnover and compromised skeletal health.

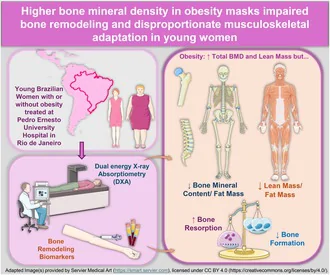

## 1 Introduction

Obesity is a disease defined as an abnormal or excessive accumulation of body fat, which is associated with the development of other disorders such as hypertension, Type 2 diabetes *mellitus* (T2DM), certain types of cancer, and osteoarthritis [1]. The expansion of white adipose tissue occurs pathologically, leading not only to an increase in fat mass but also to a profound alteration in the immune profile of the adipose tissue, turning it into an inflammatory site. As an endocrine organ, the development of obesity alters the secretory profile of white adipose tissue, thereby contributing to the onset of multiple disorders in affected individuals [2, 3].

The interaction between white adipose tissue and bone tissue is highly complex, involving both direct and indirect communication axes. Once adipose tissue acquires an inflammatory status, it can interfere with the differentiation of osteoblasts and osteoclasts, thereby disrupting the balance between these cell types during bone remodeling [4]. Other obesity‐associated mechanisms such as alterations in the gut microbiota, poor nutritional diet, the development of insulin resistance, oxidative stress [5] and physical inactivity are factors that, in the long term, contribute to bone fragility [6].

Studies using rodent models fed a high‐fat diet have demonstrated that obese animals exhibit significant trabecular and cortical bone loss of the femur or tibia [7, 8]. Histological analyses revealed that bone loss in obese animals was associated with a decrease in the number of osteoblasts [9] and a significant increase in osteoclasts [9, 10]. Measurements of serum markers of bone resorption and formation, such as carboxy‐terminal Type I collagen telopeptide (CTX1) and amino‐terminal propeptide of Type 1 procollagen (P1NP), respectively, confirm the increasing resorptive activity of osteoclasts [10, 11] and the reduced extracellular matrix synthesis activity of osteoblasts [11].

Despite the homogeneity of findings in animal models, the effect of obesity development on bone mineral density (BMD) and microarchitecture in humans remains controversial. Studies aiming to establish correlations between BMD and body mass index (BMI) or body fat percentage have reported positive correlations, suggesting that obesity exerts an osteostimulatory effect [12, 13] mainly due to the mechanical stimulus that may enhance the synthesis of bone extracellular matrix by osteoblasts.

Conversely, other studies have described negative correlations, indicating that fat mass gain is associated with the development of osteopenia or osteoporosis and an increased risk of fractures [14, 15]. This variability in results arises from the heterogeneity of study populations, as it is well known that sex, ethnicity, and age are factors directly related to bone condition [16]. Additionally, studies evaluating these parameters in women include another relevant variable: the premenopausal and postmenopausal status.

Given the broad range of confounding factors, the true impact of obesity on skeletal health is difficult to ascertain, highlighting the need for a more in‐depth analysis across different study populations. In the literature, studies evaluating BMD and bone microarchitecture in postmenopausal women with obesity are more frequent; however, aging and the decline in estrogen levels are factors that mask the contribution of adipose tissue gain to osteoporosis [17]. Moreover, the Brazilian population remains underrepresented in studies addressing this topic, despite the rising prevalence of overweight and obesity among women in Brazil [1]. In this context, assessments conducted in young Brazilian women, with or without obesity, remain valid and necessary to better understand the effects of adiposity gain on bone remodeling in a more targeted and less biased manner in a country with a mixed‐race population.

During this study we have evaluated the total BMD and the site‐specific density of the lumbar spine and femoral neck in young women with or without obesity. Additionally, we have assessed the correlations between increased adiposity, changes in lean mass, BMD, and plasmatic bone remodeling markers.

## 2 Methods

### 2.1 Ethics Statement

This cross‐sectional study was approved by the Institutional Review Board (IRB) of Pedro Ernesto University Hospital (Approval No. CAAE: 56302121.2.0000.5259). All procedures involving human participants were conducted in accordance with the ethical standards of the institutional and national research committees and with the Declaration of Helsinki and its subsequent amendments. Written informed consent was obtained from all participants prior to their enrollment in the study.

### 2.2 Participant Recruitment, Study Design, and Ethical Approval

Recruitment, pre‐participation screening, and data collection occurred between October 2024 and July 2025. A total of 54 women volunteers aged 18–40 years were included in the study. Obesity group (grades 2, 3, or 4) (*n* = 28, mean age: 32.04 ± 5.42 years) was defined as those with a BMI > 35 kg/m^2^ and a fat mass index (FMI) > 21 kg/m^2^ while the lean healthy group (*n* = 26), mean age: 29.19 ± 5.45 years by BMI of 18.5–24.9 kg/m^2^ and a FMI of 5–9 kg/m^2^. Following the initial assessment of clinical data from 158 adults, 54 participants were selected to comprise this study after the application of selection criteria (Figure 1).

**FIGURE 1:**
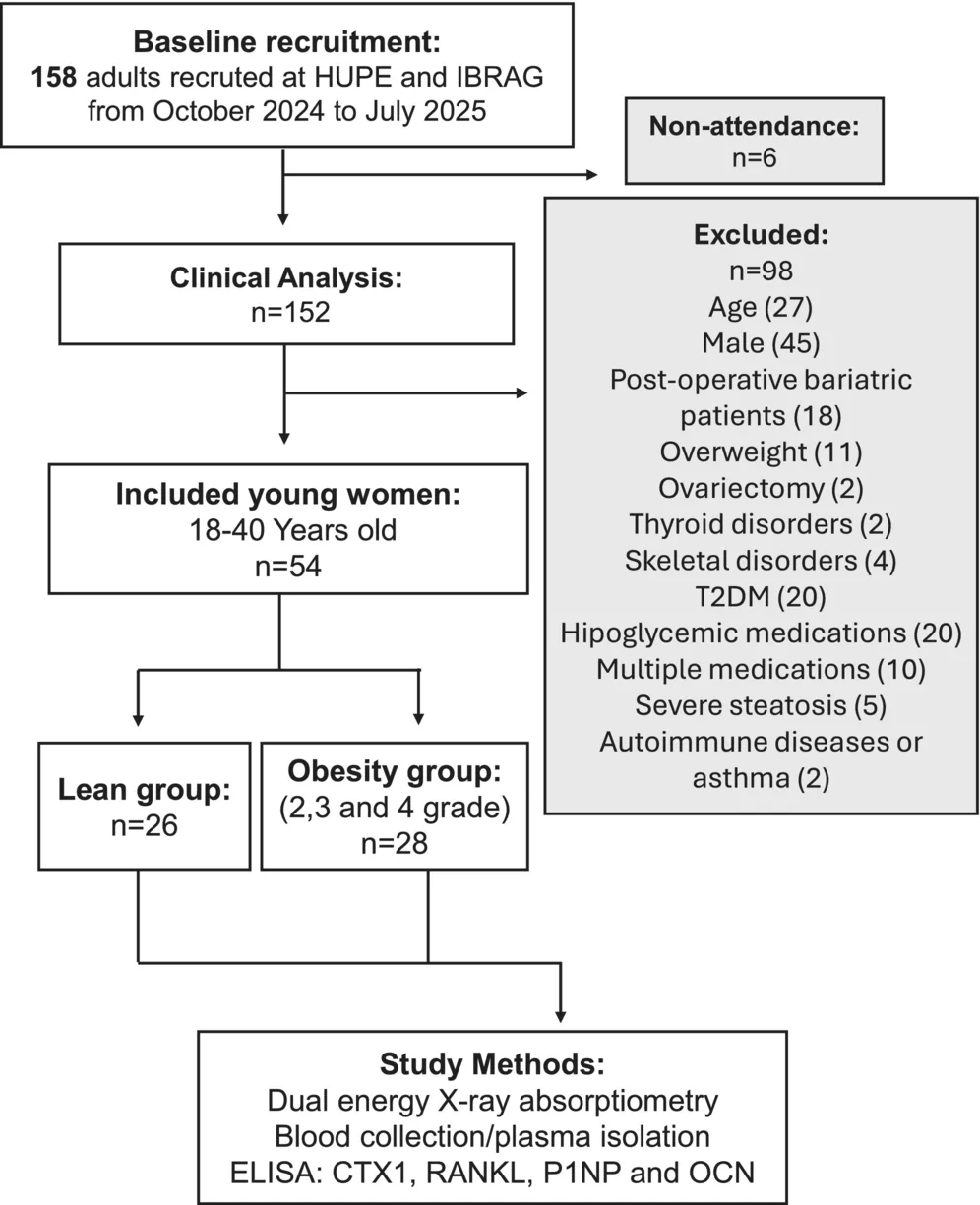
Study recruitment workflow. Following the initial assessment of clinical data from 158 adults, 54 participants were selected to comprise this study after the application of selection criteria. CTX1, Cross‐Linked C‐Telopeptide of Type I Collagen; ELISA, Enzyme‐Linked Immunosorbent Assay; HUPE, Hospital Universitário Pedro Ernesto; IBRAG, Instituto de Biologia Roberto Alcantara Gomes; OCN, Osteocalcin; P1NP, Procollagen I N‐Terminal Propeptide; RANKL, RANK ligand; TDM2, Type 2 diabetes mellitus.

The obesity group consisted of mixed‐race patients treated by the Brazilian Public Health System (SUS), eligible for metabolic and bariatric surgery, recruited from a public outpatient unit at the Pedro Ernesto University Hospital (UERJ). Within this cohort, the prevalence of comorbidities was 56.5% for hypertension, 4.3% for dyslipidemia, and 17.4% for anxiety or depressive disorders. Patients with skeletal disorders or in use of any hormonal replacement therapy or medications affecting bone metabolism, severe steatosis, thyroid disorders, T2DM (or in use of hypoglycemic medications), in use of multiple medications, ovariectomized, post‐operative bariatric patients, overweight, and males were excluded. The lean group consisted of age‐matched women with normal body weight and no history of cardiometabolic or skeletal disorders. Participants were recruited from academic community of the Institute of Biology of the UERJ (Figure 1). To minimize potential bias, biological samples were assigned coded identifiers before laboratory analyses, and investigators performing the biochemical assays were blinded to participants' group allocation. Clinical assessments and DXA measurements were conducted using standardized protocols, and statistical analyses followed a prespecified analysis plan.

### 2.3 Anthropometry and Body Composition Analysis

Body weight and height were measured using an electronic scale and stadiometer (Welmy W300A, São Paulo, SP, Brazil). BMI was calculated as body weight divided by height squared (kg/m^2^). Body composition (lean mass, fat mass, and bone mineral content) and BMD were assessed using Dual energy X‐ray absorptiometry (DXA; Lunar Prodigy Advance, GE Healthcare, Chicago, Illinois, USA). The FMI and relative skeletal muscle index were calculated by dividing the total body fat mass and lean mass of the upper plus lower limbs by height squared (kg/m^2^), respectively. BMD at the lumbar spine (L1–L4) and femoral neck was reported as absolute values (g/cm^2^) and as standard deviation scores relative to the expected BMD of an age‐matched population. BMD values were compared to the reference population using *Z*‐scores, the recommended parameter for evaluating bone mass in young women. All technical aspects of the DXA procedures were performed in accordance with current guidelines and standardized protocols [18].

### 2.4 Peripheral Blood Collection and Plasma Isolation

Peripheral blood samples (10–45 mL) were collected from each volunteer using a vacuum‐assisted collection system under aseptic conditions, performed by trained personnel. All collections were carried out in a non‐fasting state. The protective cap was removed; venipuncture was performed in 4 mL vacuum collection tubes containing K_2_EDTA anticoagulant (Cat. 360057; BD, Franklin Lakes, New Jersey, USA). Immediately after collecting, plasma was obtained by centrifuging the anticoagulated peripheral blood at 3500 rpm for 20 min at 20°C, using acceleration and deceleration setting 9 (Cat. 75004270; Thermo Fisher Scientific, Waltham, Massachusetts, USA). Following separation, the plasma fraction was carefully aliquoted into sterile, capped microtubes and stored at −80°C until subsequent biochemical analyses.

### 2.5 Enzyme‐Linked Immunosorbent Assay (ELISA)

The analytes CTX‐I, receptor Activator of Nuclear Factor‐κB Ligand (RANK‐L), and P1NP, respectively related to markers of bone resorption and bone formation, were quantified in plasma samples from all volunteers using commercial ELISA kits—Human CTX I (Cross‐Linked C‐Telopeptide of Type I Collagen) ELISA Kit (Cat. E‐EL‐H0835), Human sRANK‐L ELISA Kit (Cat. E‐EL‐H1387), and Human PINP (Procollagen I N‐Terminal Propeptide) ELISA Kit (Cat. E‐EL‐H0185) (Elabscience, Houston, Texas, USA) according to the manufacturer's instructions. Osteoprotegerin, an inhibitor of RANKL, was measured using the commercially available Human Osteoprotegerin/TNFRSF11B DuoSet ELISA kit (Cat. No. DY805) (R&D Systems Inc., a Bio‐Techne brand, Minneapolis, Minnesota, USA). Additionally, the analyte osteocalcin (OCN), a marker of bone formation, was quantified in plasma samples using the commercial Human Osteocalcin ELISA Kit (Cat. RAB1073) (Sigma‐Aldrich, St. Louis, Missouri, USA), following the manufacturer's recommendations.

### 2.6 Statistical Analysis

The experimental data was expressed as arithmetic mean ± standard deviation. The Shapiro–Wilk test was applied to each experimental group to verify data normality. For comparisons between two experimental groups, an unpaired parametric Student's *t*‐test was used. To acess the Power analysis, the effect size, which indicates the magnitude of the difference between two means, was expressed using Cohen's *d* value. Effect size magnitude was interpreted as follows: 0.2 as small, 0.5 as medium, and 0.8 as large. Other datasets were analyzed using Pearson's correlation to investigate linear relationships between two variables from the same individual. All statistical analyses and graphical representations were carried out using GraphPad Prism software, version 8 RRID:SCR_002798 (http://www.graphpad.com/) (GraphPad Software, La Jolla, California, USA). A *p*‐value of < 0.05 was considered statistically significant.

## 3 Results

### 3.1 Characterization of Fat Mass Distribution Among Volunteers

As expected, the DXA analysis of whole‐body shows that women with obesity presented greater adiposity: 53.43% of their total body mass as adipose tissue, whereas in lean women this value corresponds to 33.75%. Regarding the abdominal region, women with obesity exhibited 55.91% of trunk mass composed of fat versus 30.94% among lean volunteers. These findings lead to a mean FMI reaching 23.06 kg/m^2^ in women with obesity, compared to 7.24 kg/m^2^ in lean women. DXA analyses confirmed a markedly greater accumulation of adipose tissue across multiple body regions in women with obesity (Table 1).

**TABLE 1:** Clinical data and fat mass characterization.

	Lean women (*n* = 26)	Women with obesity (*n* = 28)	*p*	Cohen's *d*
Age (years)	29.19 ± 5.45	32.04 ± 5.42	0.07 NS	0.524
BMI (kg/m2)	22.21 ± 1.97	44.27 ± 4.99	< 0.0001****	5.813
FMI (kg/m^2^)	7.24 ± 1.58	23.06 ± 4.12	< 0.0001****	5.07
Total body fat mass (%)	33.75 ± 5.28	53.43 ± 4.20	< 0.0001****	4.13
Abdominal fat mass (%)	30.94 ± 6.75	55.91 ± 4.16	< 0.0001****	4.45

*Note:* Young volunteers with or without obesity underwent whole‐body DEXA scans to assess total fat mass. Data are expressed as mean ± standard deviation. Unpaired Student's *t*‐test and effect size calculation using Cohen's *d* (*n* = 26–28). ****p < 0.0001.

Abbreviations: BMI, body mass index; FMI, fat mass index; NS, not significant.

### 3.2 Women With Obesity Exhibit Higher BMD and Lean Mass

As shown in Table 2, whole‐body DXA analysis revealed that volunteers with obesity exhibit a higher total BMD compared to those without obesity. Supporting these findings, *Z*‐score values in the obesity group were higher (1.20) than in the lean group, reaching a mean *Z*‐score of −0.04. Complementary analysis showed an average bone mineral content (BMC) of 2.674 kg in women with obesity versus 2.135 kg of BMC in eutrophic volunteers. Accordingly, to the data obtained in the entire skeleton densitometry, the BMD of the lumbar spine (L1–L4) and the femoral neck in women with obesity was higher than those observed at the same sites in women with normal weight. The *Z*‐score values for the lumbar spine and the femoral neck were also higher in women with obesity (0.91—lumbar spine; 0.57—femur neck) compared to eutrophic women (−0.41 and −0.37, respectively).

**TABLE 2:** Muscle and skeletal changes associated with obesity.

	Lean women (*n* = 26)	Women with obesity (*n* = 28)	*p*	Cohen's *d*
Total BMD (g/cm^2^)	1.09 ± 0.09	1.22 ± 0.08	< 0.0001****	1.89
Total *Z*‐SCORE	−0.04 ± 0.97	1.20 ± 0.97	< 0.0001****	1.30
Femoral neck BMD (g/cm^2^)	0.98 ± 0.15	1.15 ± 0.12	0.0001***	1.21
Femoral neck *Z*‐SCORE	−0.37 ± 1.6	0.57 ± 1.12	0.0063**	0.83
L1–L4 BMD (g/cm^2^)	1.13 ± 0.13	1.29 ± 0.14	0.0003***	1.12
L1–L4 *Z*‐SCORE	−0,41 ± 1.13	0.91 ± 1.23	0.0003***	1.12
Total BMC (g)	2135.44 ± 247.28	2674.96 ± 309.17	< 0.0001****	1.93
Total lean mass (g)	36998.38 ± 3467.01	53704.07 ± 5335.07	< 0.0001****	3.71
RSMI (kg/m^2^)	6.21 ± 0.76	9.31 ± 0.91	< 0.0001****	3.70

*Note:* Volunteers with or without obesity underwent whole‐body DXA scans to assess BMD and lean mass. Data are expressed as mean ± standard deviation. Unpaired Student's *t*‐test and effect size calculation using Cohen's *d* (*N* = 26–28). *p < 0.05; **p < 0.01; ***p < 0.001; ****p < 0.0001.

Abbreviations: BMC, Bone mineral content; BMD, Bone mineral density; L1–L4, Lumbar spine vertebrae L1–L4; RSMI, Relative skeletal muscle index.

Skeletal muscle and bone tissues mutually regulate each other through the release of myokines and osteokines, respectively [19]. The evaluation of skeletal muscle composition of young women with obesity showed an increase in total lean mass compared to age‐matched women without obesity. The relative skeletal muscle index (RSMI) of women with obesity reached an average value of 9.31 kg/m^2^, compared to 6.21 kg/m^2^ in lean women (Table 2).

Several studies have established correlations between BMD and BMI. However, since BMI is an anthropometric measure that does not always accurately reflect adiposity status, we have opted to add other anthropometric indicators. Total skeletal BMD showed a positive association with BMI (*r* = 0.6696; *p* < 0.0001; Figure 2A), FMI (*r* = 0.6418; *p* < 0.0001; Figure 2B), and abdominal fat percent (*r* = 0.6607; *p* < 0.0001; Figure 2C).

**FIGURE 2:**
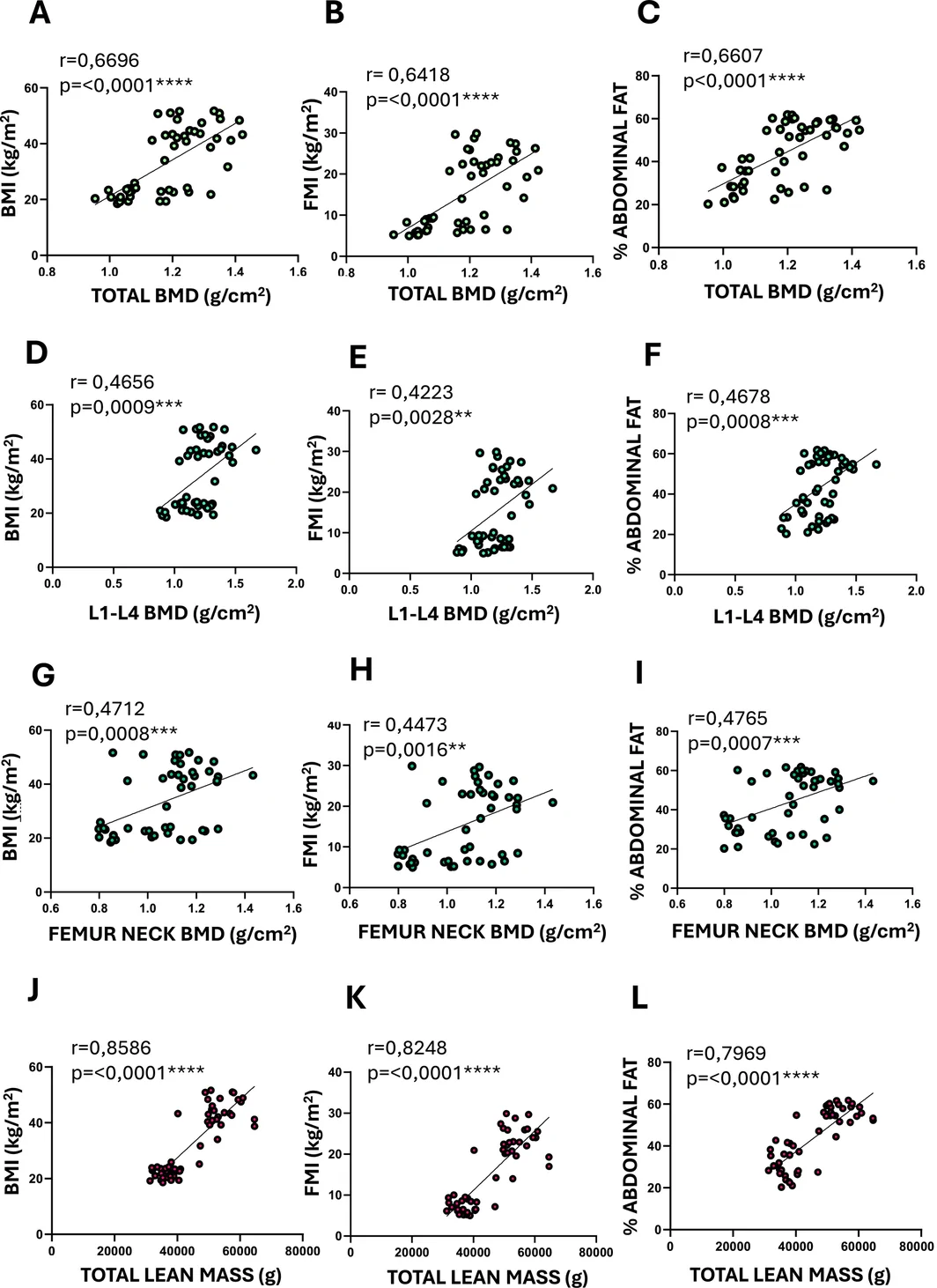
Bone mineral density and total lean mass positively correlate with obesity indicators. Volunteers with or without obesity underwent DXA scans of the whole body, lumbar spine (vertebrae L1–L4), and right femoral neck to assess bone mineral density (BMD) and fat mass. Total BMD was correlated with body mass index (BMI, kg/cm^2^) (A); fat mass index (FMI, kg/cm^2^) (B) and abdominal fat percentage (C). Lumbar spine (L1–L4) BMD was correlated with BMI (kg/cm^2^) (D); FMI (kg/cm^2^) (E) and abdominal fat percentage (F). Femoral neck BMD was correlated with BMI (kg/cm^2^) (G); FMI (kg/cm^2^) (H) and abdominal fat percentage (I). Data are presented individually. *p < 0.05; **p < 0.01; ****p < 0.0001. Pearson's correlation analysis (A–I) (*N* = 26–28).

Those patients with obesity who volunteered for the study showed a positive correlation between BMD at L1–L4 and BMI (*r* = 0.4656; *p* = 0.0009; Figure 2D), FMI (*r* = 0.4223; *p* = 0.0028; Figure 2E), and abdominal fat percent (*r* = 0.4678; *p* = 0.0008; Figure 2F). Similarly, the same pattern of associations was noticed between BMD at the femoral neck with BMI (*r* = 0.4712; *p* = 0.0008; Figure 2G), FMI (*r* = 0.4473; *p* = 0.0016; Figure 2H), and abdominal fat percent (*r* = 0.4765; *p* = 0.0007; Figure 2I). Reinforcing that the excessive mechanical load might be the primary factor driving musculoskeletal adaptations in obesity [19], we observed that total lean mass presents a strong positive association with BMI (*r* = 0.8586; *p* < 0.0001; Figure 2J), FMI (*r* = 0.8248; *p* < 0.0001; Figure 2K), and abdominal fat mass percent (*r* = 0.7969; *p* < 0.0001; Figure 2L).

### 3.3 Women With Obesity Present Osteosarcopenic State

Thus, increased body fat may initially induce adaptive responses in bone and muscle to accommodate the greater mechanical load imposed by adiposity. However, in women with obesity, muscle and bone mass increase to a lesser extent than fat mass, indicating a disproportionate musculoskeletal adaptation. Although these absolute findings may suggest an association of obesity with the formation of denser bones, the increase in BMD was not proportional to the total fat mass gain observed in women with excess weight, who showed a decreased ratio of BMC to fat mass (BMC/FM) than lean women (Figure 3A). Women with obesity had a 68.7% lower BMC/FM ratio than women in the eutrophic group. Similar to this, BMC/BMI and BMC/FMI ratios (Figure 3B,C) in women with obesity were significantly lower than those in lean women. The apparent lean mass gain in women with obesity is not observed when total lean mass was normalized by total fat mass (Figure 3D), BMI, and FMI (Figure 3E,F).

**FIGURE 3:**
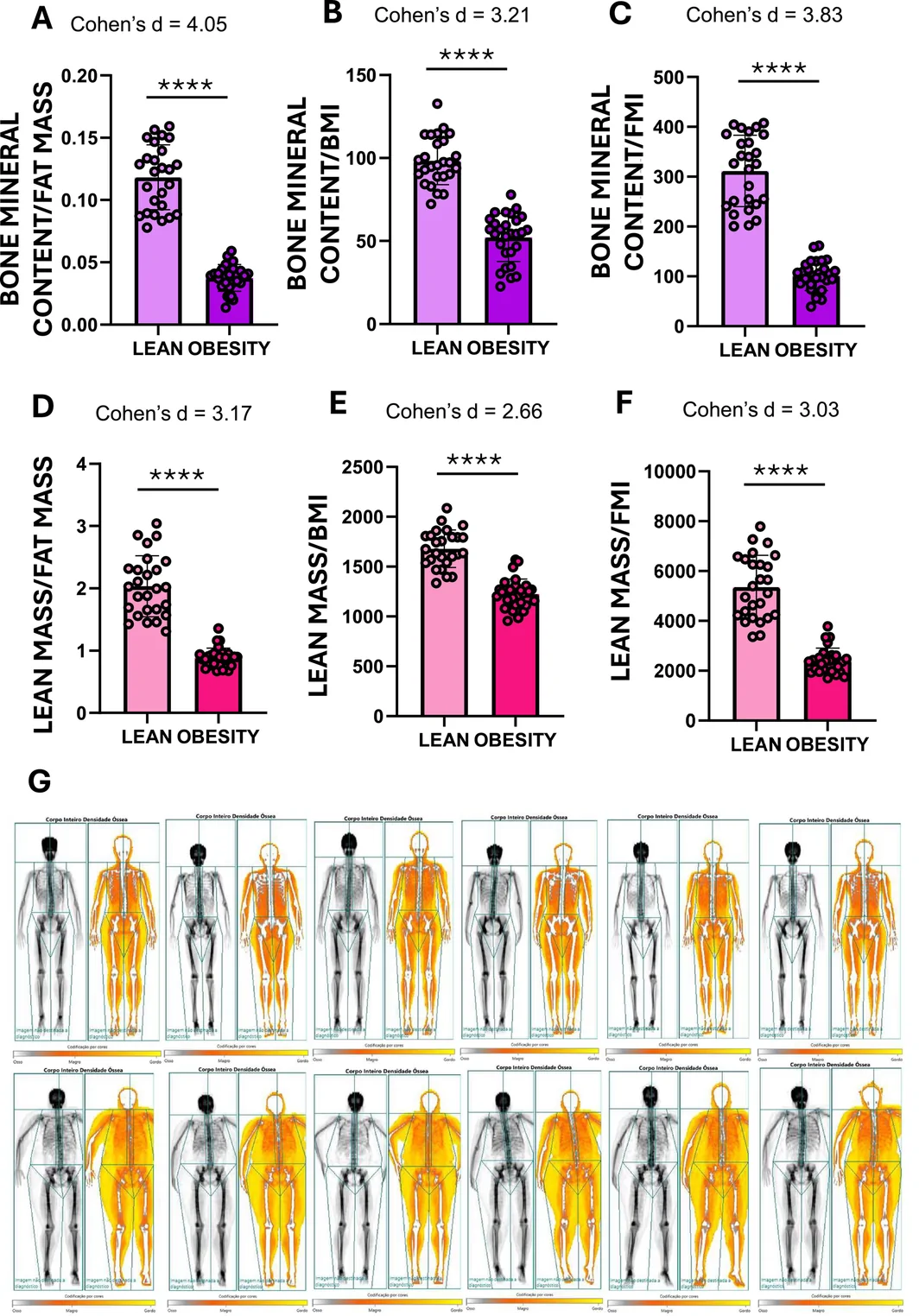
Volunteers with obesity exhibit osteosarcopenic state. The values of Bone mineral content and total lean mass from each group were normalized by total fat mass, body mass index, and fat mass index. (A) Bone mineral content and total fat mass ratio. (B) Bone mineral content and Body mass index ratio. (C) Bone mineral content and fat mass index ratio. (D) Total lean mass and total fat mass ratio. (E) Total lean mass and Body mass index ratio. (F) Total lean mass and Fat mass index ratio. ****p < 0.0001. Data are expressed as mean ± standard deviation. Unpaired Student's *t*‐test and effect size calculation using Cohen's *d*. (A–F). (G) Representatives of DXA scans (*N* = 26–28).

### 3.4 Plasma From Donors With Obesity Exhibits a Higher Concentration of the Bone Resorption Markers and Reduced Bone Formation Markers

To assess the bone osteometabolism status in volunteers with or without obesity, the bone resorption markers, CTX‐I and RANK‐L, and Osteoprotegerin (OPG), a RANK‐L inhibitor, were measured in plasma samples. Higher CTX‐I levels were observed in women with obesity compared to eutrophic volunteers (Figure 4A). CTX‐I levels showed positive correlations with BMI (*r* = 0.3651; *p* = 0.0337; Figure 4B) and FMI (*r* = 0.3663; *p* = 0.028; Figure 4C). Similarly, higher RANK‐L concentrations were observed in samples of volunteers with obesity compared to lean (Figure 4D). Plasmatic RANK‐L showed positive correlations with BMI (*r* = 0.4329; *p* = 0.0169; Figure 4E) and FMI (*r* = 0.4349; *p* = 0.0184; Figure 4F). We observed that plasma osteoprotegerin concentrations were reduced in women with obesity compared with eutrophic women (Figure 4G) and showed an inverse correlation with BMI (*r* = −0.4919; *p* = 0.0017; Figure 4H) and FMI (*r* = −0.4669; *p* = 0.0031; Figure 4I). Thus, the increased RANKL/OPG ratio observed in women with obesity (Figure 4J) suggests a shift toward a more osteoclastogenic profile, as further supported by its positive correlations with BMI (*r* = 0.3717; *p* = 0.0395; Figure 4K) and FMI (*r* = 0.3653; *p* = 0.0433; Figure 4L).

**FIGURE 4:**
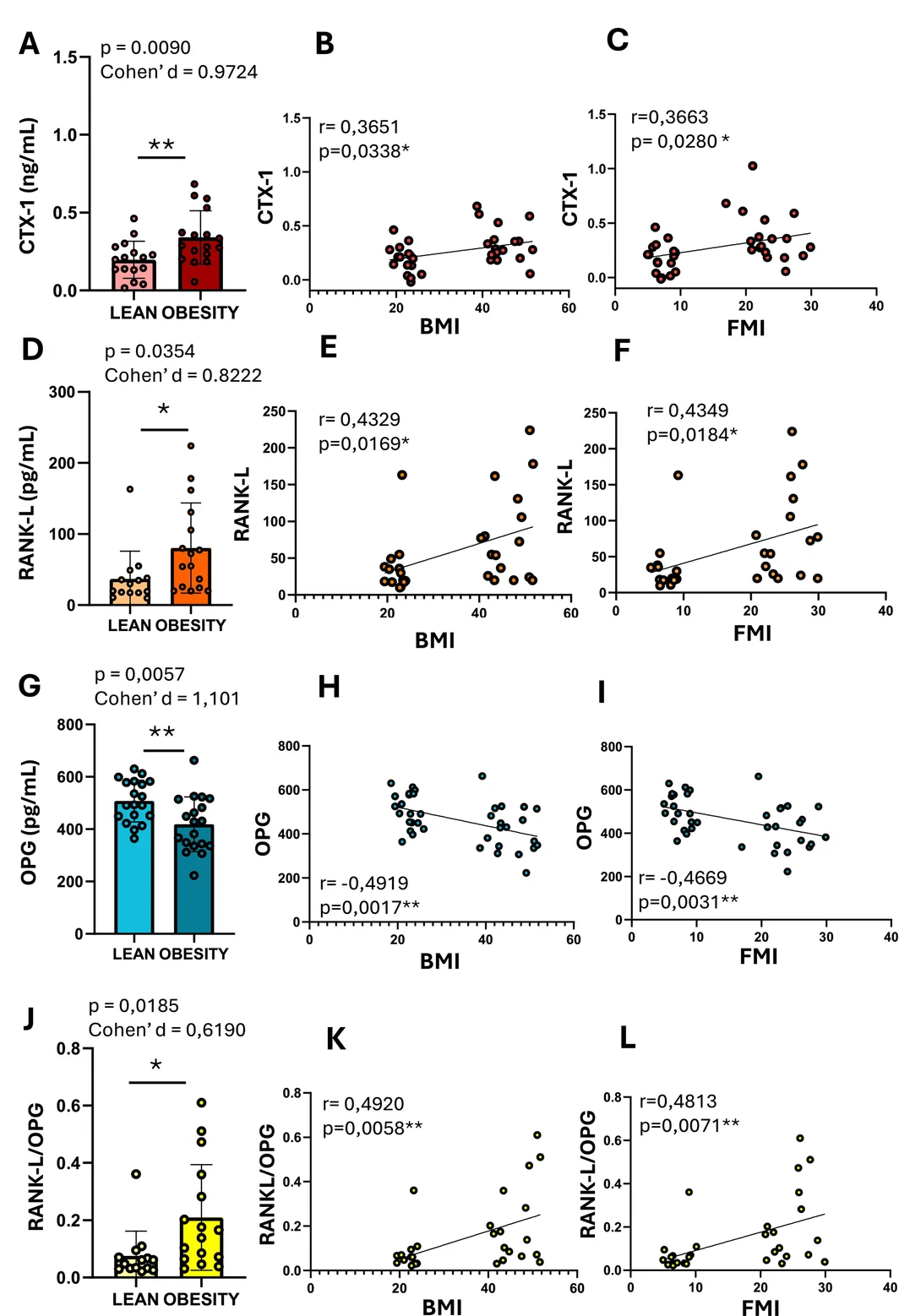
Plasma from donors with obesity exhibits a higher concentration of bone resorption markers. Plasma samples from young women volunteers with or without obesity were collected for the quantification of the bone resorption markers CTX‐1 and RANK‐L and RANK‐L inhibitor Osteoprotegerin (OPG). (A) Quantification of C‐terminal telopeptide of Type I collagen (CTX‐1) expressed in ng/mL. CTX‐1 levels were correlated with body mass index (BMI, kg/cm^2^) (B) and fat mass index (FMI, kg/cm^2^) (C). (D) Quantification of receptor activator of nuclear factor kappa‐B ligand (RANK‐L) expressed in pg/mL. RANK‐L levels were correlated with BMI (kg/cm^2^) (E) and FMI (kg/cm^2^) (F). (G) Quantification of Osteoprotegerin (OPG) expressed in pg/mL. OPG levels were correlated with body mass index (BMI, kg/cm^2^) (H) and fat mass index (FMI, kg/cm^2^) (I). (J) RANK‐L/OPG ratio was calculated. RANK‐L/OPG levels were correlated with BMI (kg/cm^2^) (K) and FMI (kg/cm^2^) (L). Data are expressed as mean ± standard deviation or as individual values. *p < 0.05; **p < 0.01; NS—not significant. Unpaired Student's *t*‐test and effect size calculation using Cohen's *d*. (A, D, G, J). Pearson's correlation analysis (B, C; E, F; H, I; K, L) (*N* = 17–19) for CTX‐1 and OPG analysis. (*N* = 14–16) for RANK‐L and RANK‐L/OPG analysis.

Additionally, a significant reduction was observed in the P1NP (Figure 5A) and OCN (Figure 5D) plasmatic levels in volunteers with obesity compared to lean. Noteworthy, plasma P1NP levels were negatively associated with BMI (*r* = −0.499; *p* = 0.0036; Figure 5B) and FMI (*r* = −0.4596; *p* = 0.0081; Figure 5C). Similarly, negative correlations were found between OCN and BMI (*r* = −0.4654; *p* = 0.0048; Figure 5E) and FMI (*r* = −0.4916; *p* = 0.0031; Figure 5F). All these data demonstrate that obesity development promotes an altered bone remodeling, with increased osteoclast activity and inhibition of osteoblast function.

**FIGURE 5:**
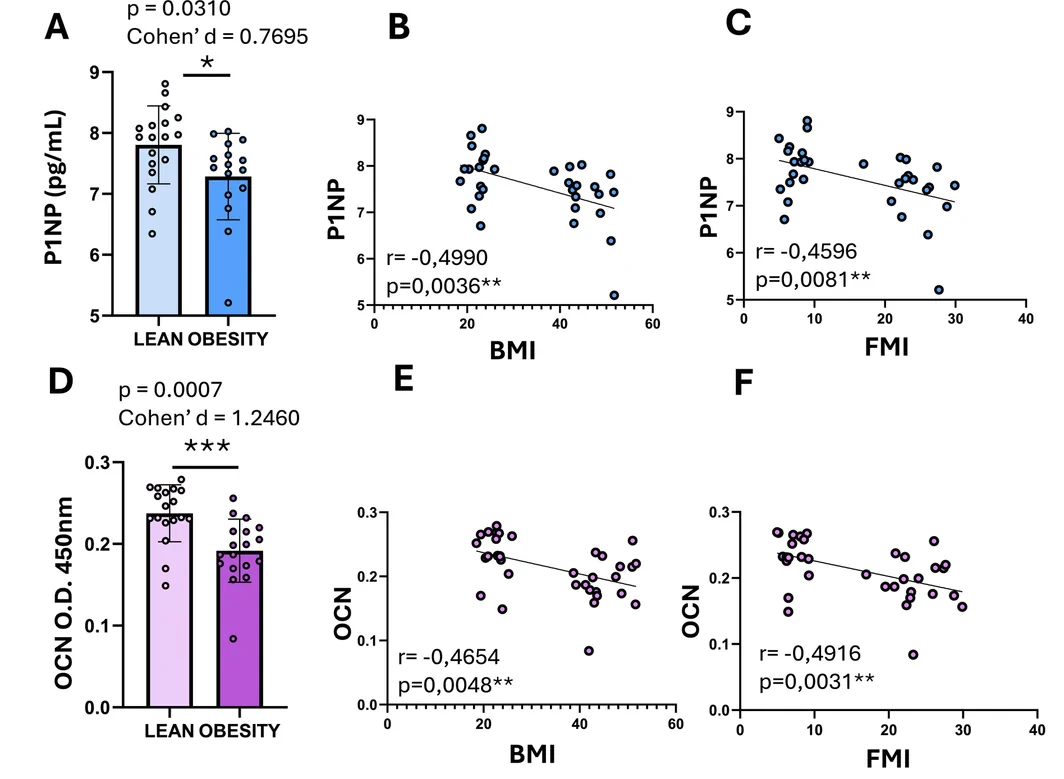
Plasma from donors with obesity exhibits reduced concentration of formation markers. Plasma samples from young women with or without obesity were collected for the quantification of the bone formation markers P1NP and osteocalcin (OCN). (A) Quantification of the N‐terminal propeptide of Type I procollagen (P1NP) expressed in ng/mL. P1NP levels were correlated with body mass index (BMI, kg/cm^2^) (B) and fat mass index (FMI, kg/cm^2^) (C). (D) Quantification of osteocalcin (OCN) expressed as optical density (O.D.) at 450 nm. OCN levels were correlated with BMI (kg/cm^2^) (E) and FMI (kg/cm^2^) (F). Data are expressed as mean ± standard deviation or as individual values. *p < 0.05; *p* < 0.01; ***p < 0.001; NS, not significant. Unpaired Student's *t*‐test and effect size calculation using Cohen's *d*. (A, D). Pearson's correlation analysis (B, C; E, F) (*N* = 17–19).

## 4 Discussion

To assess the impact of obesity on body composition in young Brazilian women, we examined the potential correlations between fat mass, lean mass, BMD, and plasma markers of bone formation and resorption. Our data demonstrated that total and site‐specific BMD in women with obesity were higher than in lean women, as mirrored by the *Z*‐score values. In this context, different anthropometric measures, such as BMI and FMI, showed positive correlations with BMD. Total and abdominal fat mass were positively associated with increases in total, femoral neck, and lumbar spine BMD. Notably, this positive correlation was also observed between upper‐ and lower‐limb BMD and the fat mass of each respective segment, supporting the idea that in young women, fat mass gain contributes to increased bone mineralization through mechanical loading stimulation.

Osteogenesis is reported to be stimulated during the application of constant mechanical loading [6]. Thus, the considerable gain of total and regional adipose tissue during obesity can initially act as an additional mechanical effort for the skeleton, promoting increased BMC. Nevertheless, the gain in BMD is not necessarily associated with an increase in bone strength. Healthy bone remodeling depends on the balanced deposition of organic and inorganic matrix components; excessive mineralization may render bones extremely rigid and more susceptible to fractures.

In accordance with our findings, MAΪMOUN et al. demonstrated that, in a cohort of French women aged 18–35 years, total BMD and *Z*‐scores, as well as the BMD of specific sites such as the lumbar spine, hip, and radius, were higher in women with obesity compared to eutrophic ones. In another study, younger women aged 16–27 years with obesity presented increased lumbar spine and radial BMD and *Z*‐scores [20]. Similarly, the authors also showed a positive association of BMD with BMI and total fat mass [20, 21].

Other studies have examined the degrees of obesity severity among young women, excluding lean individuals. Lebanese women aged 19–47 years were subdivided into groups with obesity II (BMI 35–39.9), obesity III (BMI 40–50), and obesity IV (BMI > 50), and BMD analyzed by DXA. Supporting the hypothesis that an increase in inorganic bone fraction is not necessarily indicative of improved bone condition, it was observed that women with obesity IV had higher bone mineral content than the other groups yet exhibited the lowest femoral neck strength index [22].

Few studies have specifically addressed young women from a mixed‐race country like Brazil, while others involving postmenopausal women are far more abundant. In this context, the literature presents divergent findings regarding the effects of fat mass gain on BMD. Yuan et al. [23] evaluated 564 postmenopausal women of various ethnicities and observed positive associations between BMD parameters (total femur, femoral neck, and lumbar spine) and anthropometric measures (BMI and waist circumference), without assessing bone strength or resistance. Nevertheless, the work by JOSE et al. revealed that the mean femoral neck BMD of women with severe obesity was lower than that of age‐matched women with moderate obesity. Furthermore, evaluation of the trabecular bone score indicated that women with severe obesity presented the lowest values for this parameter when compared to non‐obese and moderately obese women [24].

Altogether, the data reinforces that the impact of obesity development on body composition is highly complex and strongly influenced by age. Then, it is conceivable to propose that, in younger women, fat mass gain initially exerts a mechanically osteostimulatory effect that outweighs the deleterious effects promoted by adipose tissue secretion. However, as obesity severity and chronicity increase, combined with the natural hormonal decline of aging in women, the detrimental influence of pathological adipose tissue secretion becomes predominant, leading to bone loss.

Despite the increase in BMD observed in young brazilian women in the present study, our data revealed that volunteers with obesity exhibited higher concentrations of the bone resorption markers CTX‐1 and RANK‐L/OPG and lower concentrations of the bone formation markers P1NP and OCN compared to lean women. These findings suggest that the high BMD values are not accompanied by a healthy bone remodeling in women with obesity. That imbalance in bone metabolism can lead toward bone loss in the future. In agreement with our data, MACIEJEWSKI and colleagues showed that plasma P1NP concentration was decreased in premenopausal women with obesity, without significant differences in CTX‐1 concentration [25]. Similarly, plasma OCN and CTX‐1 levels were reduced in premenopausal women with obesity compared to lean women. A tendency toward decreased P1NP levels was also reported, indicating impaired tissue turnover in obesity [20].

In a previous study, we demonstrated that alterations in bone cell activity are modulated by the secretory activity of adipose tissue in obesity. Comparable to the reduced levels of bone formation markers produced by osteoblasts observed in women with obesity shown herein, the treatment of osteoblast‐like cells, in vitro, with the adipose tissue secretome derived from women with obesity induced a loss of maturity, impairing their capacity to form a calcified extracellular matrix. We found that excess TGF‐β1 produced by adipose tissue in obesity inhibited osteoblast activity in vitro [26]. Mediators released by adipose tissue are not restricted to the local tissue and may become systemically available. Therefore, it is feasible that, besides TGF‐β1, other mediators released by obese adipose tissue may interfere in osteoclastogenesis, supporting increasing bone resorption. Pro‐inflammatory adipokines and free fatty acids may also contribute to the alteration of bone remodeling [5].

Leptin is a hormone that regulates satiety and plays an important role in bone remodeling. Evidence has shown, for example, that leptin‐knockout rodents develop obesity and exhibit increased bone mineral density in the lumbar region, but reduced femoral [27] and tibial bone mineral density [28]. In obesity, both in rodent models and in humans, dysfunctional white adipose tissue increases its capacity to produce leptin [2]. This hormone is initially thought to inhibit osteoclast differentiation and also exerts a direct osteostimulatory effect. Despite this modulation, which may appear beneficial to bone mass at first glance, obesity is characterized by leptin resistance, thereby potentially interfering with its osteoprotective effects. Additionally, adipose tissue in obesity reduces the production of adiponectin, a hormone involved not only in improving glucose metabolism but also in reducing osteoclastogenesis and promoting osteogenic differentiation, as well established in the literature [5]. We believe that reduced adiponectin levels and leptin resistance may partially explain the alterations in bone remodeling observed in young women with obesity in our study.

Additionally, lipid transport‐related proteins such as apolipoprotein E (APOE) have been reported to promote osteoblast activity while reducing osteoclast activity. Interestingly, APOE knockout in young rodents promotes a degree of bone mass gain, whereas in older rodents it produces the opposite effect [29]. Thus, the increased circulating fatty acid levels observed in women with obesity (data not shown) are consistent with our findings, indicating that despite an immediate increase in bone mass, these women already exhibit alterations in bone tissue remodeling.

In agreement with our findings, the study by MAΪMOUN and colleagues also observed that BMI and fat mass were associated with an increase in total, trunk, and appendicular lean mass, but with a decrease in the appendicular muscle mass‐to‐BMI ratio, an indicator of sarcopenia. Conversely, in women with obesity over 60 years of age, the appendicular muscle mass‐to‐BMI ratio, but also total, trunk, and limb lean mass, were lower than in young women with obesity [21].

However, an increase in lean mass does not necessarily imply an improvement in functional parameters, as occurs for BMD. The rise in lean mass in obesity has been identified as an indicator of pathological conditions. The increase of the lean mass in obese children and adolescents [30] has been shown as a predictor of left ventricular hypertrophy and a factor associated with atrial fibrillation with a correlation stronger than those observed for adiposity‐related parameters [31].

In this study we sought to correlate BMD, lean mass, and bone remodeling plasmatic markers with more accurate indicators of obesity, including not only BMI but also the FMI and the percentages of total and abdominal fat mass. The inclusion of young women as the study population eliminates the potential interference of aging on the musculoskeletal condition of the volunteers, thereby providing a more accurate representation of the alterations induced by excessive adiposity. Nevertheless, as a significant limitation, we identify the lack of assessment of bone microarchitecture patterns among volunteers with and without obesity. Those parameters could better support the hypothesis that the increase in BMD associated with greater adiposity may be more deleterious than osteoprotective.

Physical activity and dietary intake were not specifically assessed in the present study. Although women with obesity routinely received nutritional and physical activity counseling as part of their clinical care, adherence to these recommendations was not quantified, and the same limitation applies to adherence to physical activity recommendations among control participants. Therefore, the potential influence of physical activity and dietary habits on the study outcomes cannot be ruled out.

Although blood samples were not collected under fasting conditions, blood collection was standardized between 9:00 and 10:00 AM for both groups, in accordance with recommendations for the assessment of biochemical markers of bone remodeling and to minimize circadian variability [32]. Blood collection was performed after routine clinical consultations in the bariatric surgery service, as collection immediately before anesthesia on the day of surgery was not feasible due to the need to prioritize the hospital's surgical workflow. To improve comparability between groups, blood samples from control participants were collected under the same non‐fasting conditions. However, postprandial effects could not be completely excluded and may contribute to variability in CTX‐I levels. Therefore, this limitation should be considered when interpreting the present findings, and future studies should preferably standardize both fasting status and blood collection time.

In conclusion, weight and fat mass gain are associated with increased total and site‐specific BMD, as well as with increased lean mass in young Brazilian women with obesity. However, despite this apparent benefit of obesity on BMD in these volunteers, higher plasmatic CTX‐1 and RANK‐L/OPG and lower P1NP and OCN, which respectively are related to bone resorption and formation, indicate impaired bone remodeling. Thus, the gain in BMC and lean mass observed in young women with obesity may be interpreted as deleterious to the musculoskeletal system, and the persistence of obesity throughout these patients' lives is likely to result in worst bone outcomes.

## Author Contributions

Conceptualization: Y.S.F. and C.B.‐F. Methodology: Y.S.F., C.A.‐S.; C.P.‐M.; L.G.‐J.; A.P.‐D.‐C.; K.G.L.; L.G.K.‐A., and P.R.F.L. Investigation: Y.S.F. Formal Analysis: Y.S.F. Data Curation: Y.S.F. Writing – Original Draft: Y.S.F. Writing – Review and Editing: Y.S.F.; K.G.L.; L.G.K.‐A.; P.R.F.L., and C.B.‐F. Supervision: K.G.L.; L.G.K.‐A., and C.B.‐F. Funding Acquisition: C.B.‐F.

## Funding

This research was funded by: (1) National Council for Scientific and Technology Development—CNPq/Ministry of Science, Technology and Innovation, Brazil, grants #305737/2021‐0; #402918/2023‐1; #444675/2023‐0 (DECIT‐MS); (2) Foundation for the Support of Scientific Research of the State of Rio de Janeiro (FAPERJ), Rio de Janeiro State Government, grants #E‐26/204.007/2024; #E‐26/210.065/2023. This study was also funded by graduate research scholarships from the Coordination for the Improvement of Higher Education Personnel (CAPES/PROEX) of the Brazilian Ministry of Education.

## Ethics Statement

This cross‐sectional study was approved by the Research Ethics Committee of the Pedro Ernesto University Hospital (HUPE) under protocol CAAE: 56302121.2.0000.5259.

## Conflicts of Interest

The authors declare no conflicts of interest.

## Data Availability

The data underlying this article are available in *Figshare* at https://figshare.com/s/4be94d5582a7bb0e5e21. doi:10.6084/m9.figshare.31566784.
